# Noninvasive intracranial pressure monitoring in women with migraine

**DOI:** 10.1038/s41598-022-06258-9

**Published:** 2022-02-16

**Authors:** Denise Martineli Rossi, Débora Bevilaqua-Grossi, Sérgio Mascarenhas, Hugo Celso Dutra de Souza, Gabriela Ferreira Carvalho, Ana Carolina Carmona Vendramim, Stella Vieira Philbois, Fabíola Dach, Francisco José Tallarico, Anamaria Siriani de Oliveira

**Affiliations:** 1 Ribeirão Preto Medical School, Department of Health Sciences, University of São Paulo, Ribeirão Preto, São Paulo Brazil; 2 Department of Applied Physiotherapy, Federal University of Triângulo Mineiro, Uberaba, Minas Gerais Brazil; 3 São Carlos Institute of Physics, University of São Paulo, São Carlos, Brazil; 4 Institut für Gesundheitswissenschaften, Studiengang Physiotherapie, Pain and Exercise Research Luebeck (P.E.R.L), Universität zu Lübeck, Lübeck, Germany; 5 Department of Neurosciences and Behavioral Sciences, Ribeirão Preto Medical School, University of São Paulo, Ribeirão Preto, Brazil; 6 Braincare Desenvolvimento e Inovação Tecnológica S.A., São Carlos, Brazil

**Keywords:** Chronic pain, Outcomes research

## Abstract

This cross-sectional study aimed to compare the waveform morphology through noninvasive intracranial pressure (ICP-NI) measurement between patients with migraine and controls, and to analyze the association with clinical variables. Twenty-nine women with migraine, age 32.4 (11.2) years and headache frequency of 12.6 (7.5) days per month and twenty-nine women without headache, age 32.1 (9.0) years, were evaluated. Pain intensity, migraine disability, allodynia, pain catastrophizing, central sensitization and depression were evaluated. The ICP-NI monitoring was performed by a valid method consisting of an extracranial deformation sensor positioned in the patients’ scalp, which allowed registration of intracranial pressure waveforms. Heart rate and blood pressure measurements were simultaneously recorded during 20 min in the supine position. The analyzed parameter was the P2/P1 ratio based on mean pulse per minute which P1 represents the percussion wave related to the arterial blood pression maximum and P2 the tidal wave, middle point between the P1 maximum and the dicrotic notch. There was no between-groups difference in the P2/P1 ratio (mean difference: 0.04, IC95%: -0.07 to 0.16, *p* = 0.352, F (1,1) = 0.881) adjusted by body mass index covariable. The Multiple Linear Regression showed non-statistical significance [F (5,44) = 1.104; *p* = 0.372; R^2^ = 0.11)] between the P2/P1 ratio and body mass index, presence of migraine, central sensitization, pain catastrophizing and depression. We found no correlation (*p* > 0.05) between P2/P1 ratio and migraine frequency, migraine onset, pain intensity, pain intensity at day of examination, disability, allodynia. Migraine patients did not present alterations in the waveform morphology through ICP-NI compared to women without headache and no association with clinical variables was found.

## Introduction

A central nervous system sensitization has been associated with the experience of persistent pain and symptoms such as hyperalgesia, allodynia and hypersensitivity, due to sensory processing changes, neuroplasticity and neuroinflammation^1,2^. Studies have also suggested a possible association between these symptoms with the increased intracranial pressure (ICP) in conditions such as chronic fatigue syndrome, fibromyalgia, unexplained widespread pain^3,4^ and also in migraine^5^. The hypotheses point to an unknown effect of cerebrospinal fluid pressure dysregulation and ICP that could have harmful effects on the central nervous system, brain and peripheral nerves comparing with the harmful effects of increased blood pressure^4^.

The role of increased cerebrospinal fluid pressure has already been investigated in patients with migraine^5–7^. Migraine is defined as a primary brain disorder and affects about 12% of the population with a peak prevalence between 22 and 55 years, being more prevalent in females (17%) compared to males (6%). The pathophysiology of migraine comprises a complex neurovascular dysfunction involving changes in brain excitability, inflammation of neurogenic origin with consequent vasodilation of cerebral blood vessels, in addition to recurrent activation and sensitization of trigeminovascular system pathways^8–10^. Neurogenic inflammation in the blood vessels of the meninges, in the large cerebral arteries and in the sinuses, may be responsible for sending nociceptive afferents, through mechanical, electrical or chemical stimuli, giving rise to a unilateral, pulsatile headache associated with neurological symptoms such as hypersensitivity to light, sound, smell and nausea^8,9^. The clinical manifestations of migraine differentiate it from other headaches, and the onset of a migraine crisis is often associated with triggering factors such as stress, hormonal fluctuation (menstrual period), sleep disorders, sensory overload (odors or excessive light)^8^. In addition, intrinsic triggering factors such as vascular mechanisms are also suggested, however they need to be further investigated^9^.

An investigation of the cerebrospinal fluid pressure opening pressure in 98 patients with migraine and chronic tension-type headache by an invasive monitoring showed that 55% of patients had normal cerebrospinal fluid pressure (< 200 mmH20) and normal mean waveform pressure over one-hour monitoring^6^. However, 27% had normal opening pressure and elevated mean cerebrospinal fluid pressure (> 200 mmH20) associated with abnormal waveforms and 18% had elevated opening pressure and elevated mean cerebrospinal fluid pressure (> 200 mmH20) also associated with abnormal waveforms^6^. The abnormal pressure waveforms during one-hour monitoring were more frequent in patients with chronic migraine and bilateral transverse sinus stenosis diagnosed on cerebral magnetic resonance venography^6^. However, despite the aforementioned evidence and the recently raised hypotheses of changes in pressure of cerebrospinal and intracranial pressures in migraine patients, these alterations have not been further investigated considering the risks of the invasive methods of the intracranial pressure measurement.

Non-invasive modalities for intracranial pressure monitoring have currently been investigated and proposed in the literature due to the fact that invasive methods, considered as the gold standard, present risks of infection, hemorrhage, require anesthesia, and are expensive^11^. Some proposed methods consider indirect anatomical or physiological measures that can be influenced by changes in intracranial pressure and include computed tomography, magnetic resonance imaging, transcranial Doppler ultrasound and infrared spectroscopy^11^. Another non-invasive method was recently developed and validated^12–14^. This method considers an extracranial strain gauge sensor that, when positioned in the frontolateral region, detects small variations in cranial deformation induced by intracranial pressure waveforms^14,15^. This Food and Drug Administration (FDA)-cleared non-invasive sensor and software to record the variations induced by intracranial pressure waveforms and convert them into numerical values that can be interpreted in real time or analyzed later^14,15^. This method was validated in a set of neurocritical patients^14,16^, including traumatic brain injury, subarachnoid hemorrhage and stroke, by correlating the biometric amplitude ratio from non-invasive with the ICP waveform morphology from invasive catheter monitoring (area under the receiver operator curve [AUROC], 0.9)^14^. Also, it has been recently shown a strongly positive correlation of cerebrospinal compliance measurement comparing this noninvasive intracranial pressure (ICP-NI) measurement with a transcranial Doppler blood flow analysis in hydrocephalus patients^17^.

Intracranial pressure analysis in time domain reveals a waveform morphology with three main peaks: the P1 component (percussion wave) that reflects the ejection of blood from the heart transmitted by the intracranial arteries and the choroid plexus; the P2 component (repercussion wave) that reflects cerebral compliance; and the P3 component (dicrotic wave) that represents the closure of the cardiac aortic valve^18^. Under normal physiological conditions, the waveform morphology reveals a pattern in which P1 > P2 > P3. Changes in the waveform morphology may reveal changes in brain compliance, in which P2 > P1 shows reduced brain compliance^19–21^. Thus, the aim of this study was to investigate possible alterations in the waveform morphology through ICP-NI measurement in patients with migraine and its correlation with clinical variables. We hypothesized an increased ratio (P2/P1), i.e., P2 higher than P1, in patients with migraine and correlation between the P2/P1 ratio and clinical variables.

## Methods

### Participants

This cross-sectional study evaluated 58 participants including women with migraine (n = 29) and headache-free women as a control group (n = 29). The patients with migraine were recruited from a tertiary headache clinic in Ribeirão Preto, Brazil. Inclusion criteria were: women aged 18–50 years, diagnosis of migraine by an experienced neurologist according to the International Classification of Headache Disorders (ICHD-III)^22^. Exclusion criteria were: diabetes mellitus, gestational period, diagnosis of idiopathic intracranial hypertension (according ICHD-III), concomitant primary headaches other than migraine, cardiovascular diseases, habitual use of corticosteroids, history of alcohol abuse in the last 6 months and body mass index higher than 30 kg/m2. The control group was recruited using convenience sampling and the age-matched women were included if they had no current or past diagnosis of any primary headache, no history of recurrent secondary headaches within the last 6 months which required treatment. We included those with an occasional headache, such as headache related with a flu or after alcohol exposure etc. To be included, the control participants also had to have less than 30 points in the Part A of the Central Sensitization Inventory^23^.

### Experimental procedure

The experimental procedure was performed in one laboratory visit. All participants were evaluated during the afternoon and all women were in the follicular phase of the regular menstrual cycle at the time of evaluation, i.e. up to 14 days after the beginning of the menstrual cycle^24^. Participants were asked to avoid drinking caffeine and alcohol and not to perform intense physical activity 24 h before the test. Initially, anthropometric and sociodemographic data and self-reported variables were collected. Then, the noninvasive intracranial pressure measurement (ICP-NI) was continuously and simultaneously collected with the blood pressure by a plethysmography recording equipment during 20 min, after 10 min-resting, in the supine position. The total experimental procedure lasted around 60 min. All methods were performed in accordance with relevant guidelines and regulations.

### Clinical assessment

Clinical characteristics including the frequency of migraine attacks, migraine onset and attack duration (hours) were recorded in the migraine group. Chronic migraine was defined as having attacks more than 15 days per month and episodic and episodic migraine as having at least three migraine attacks per month, according to the IHCD-III. The numeric pain rating scale (range 0–10), the Migraine Disability Assessment (MIDAS)^25^ and the 12-item Allodynia Symptom Checklist^26^ were used to evaluate the pain intensity, disability and allodynia severity. The Pain Catastrophizing Scale^27^, the Beck Depression Inventory^28^ and the Central Sensitization Inventory-BR^23^ were also administrated to characterize the samples.

### Instrumentation

**Figure 1:**
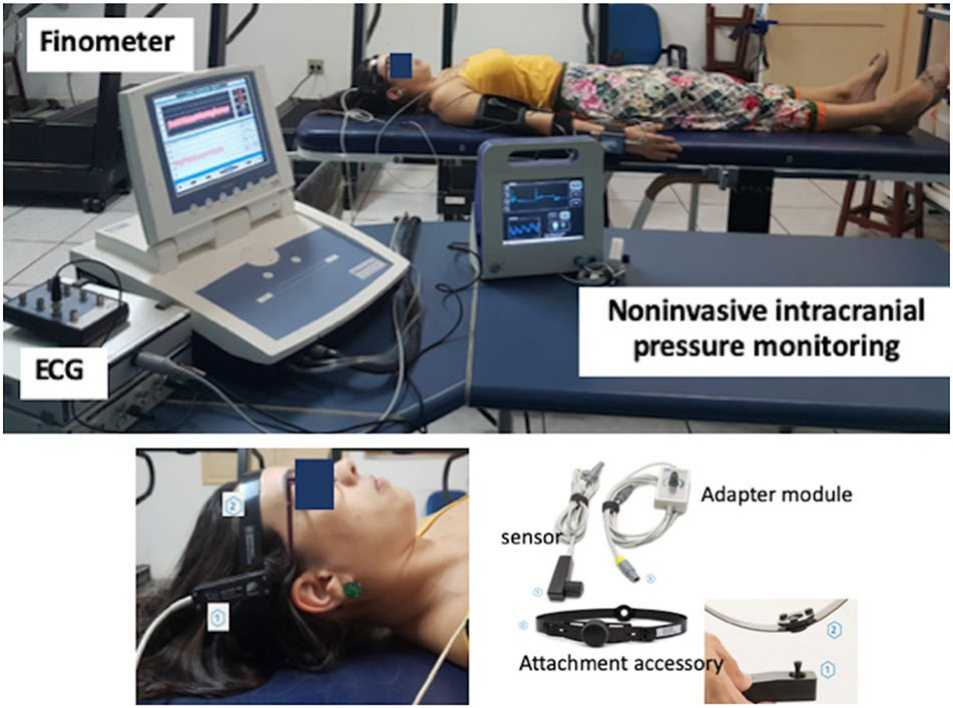
Illustration of the data collection and the positioning of the extracranial sensor.

The ICP-NI monitoring was performed using an extracranial strain gauge sensor positioned in the frontolateral region to detect small variations in cranial deformation induced by intracranial pressure waves^12,13^. The measurements were obtained by a hardware Braincare® 2.0 e by a software (Braincare® 2014.02) (Fig. 1).

**Figure 2:**
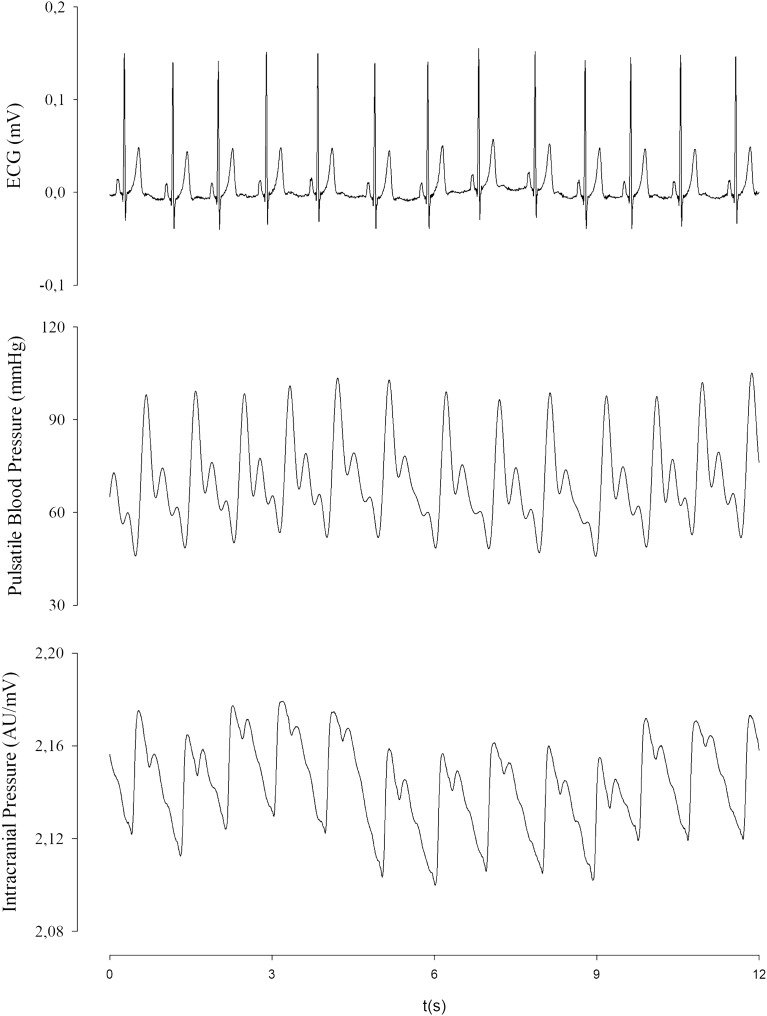
Illustration of the data synchronization of heart rate (mV), blood pressure (mmHg) and the ICP-NI measurement (mV).

This measurement was continuously and simultaneously collected with the blood pressure by a plethysmography recording equipment (Finometer Pro, Finapres Medical System, Amsterdam) and an electrocardiogram digital recorder (Dual Bio Amp/Stimulator; ADInstruments) during 20 min in the supine position, after 10 min-resting. The data were recorded by an interface to the microcomputer by using the PowerLab4/35 device (ADinstruments) and a software for data analysis (Sofware LabChart 7.0; ADInstruments) (Fig. 2).

### Data analysis

**Figure 3:**
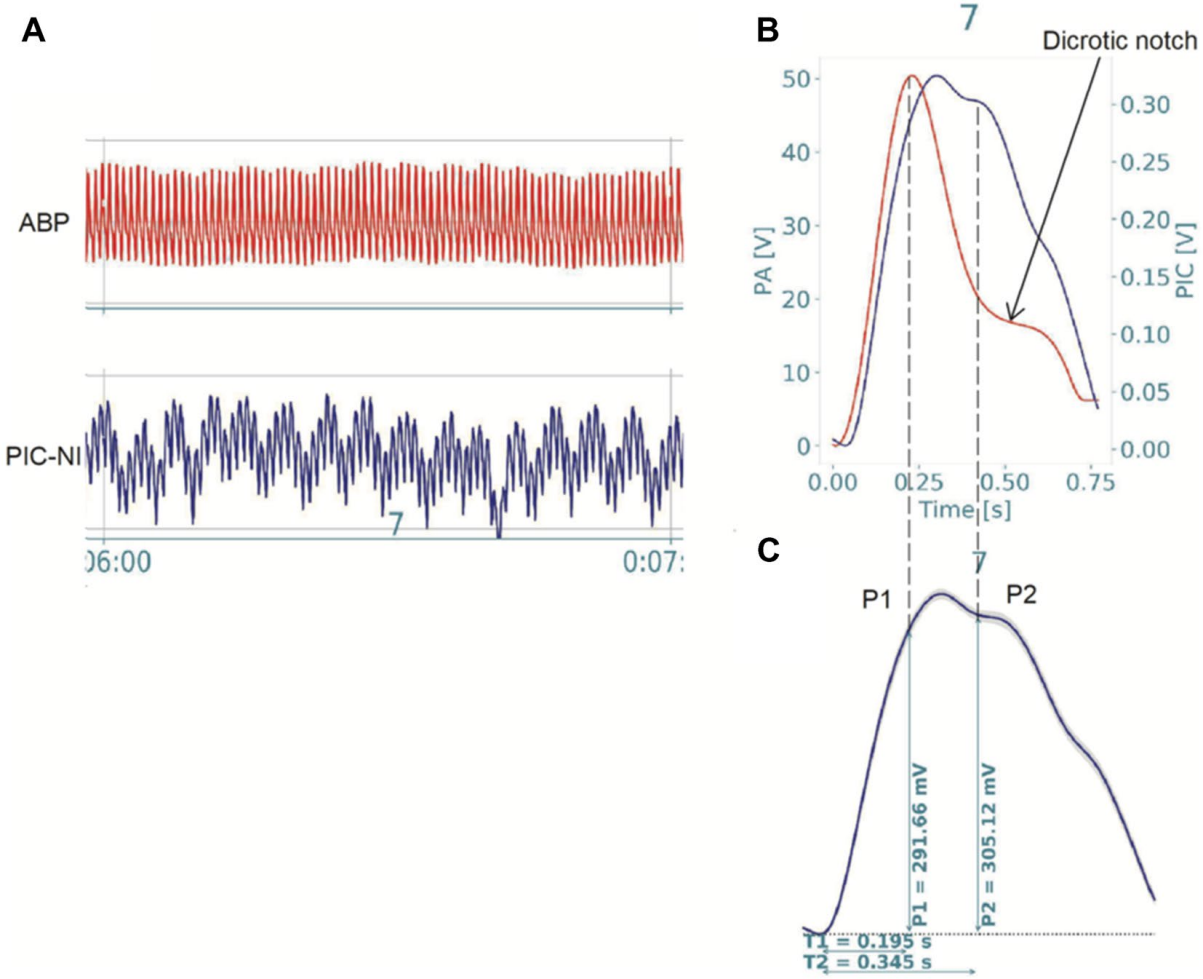
Illustration from a representative migraine patient. (**A**) Arterial blood pressure (ABP) and noninvasive intracranial pressure (ICP-NI) from minute seven of data collection. (**B,C**) Mean pulse of ABP and ICP-NI synchronized data illustrating the arterial blood pression maximum and the dicrotic notch and the P1 and P2 corresponding values and ratio.

The signal processing from the intracranial pressure data was performed by custom programs written in python and the mean pulse per minute was calculated (Fig. 3-A). Some parameters based on pulse waveform were estimated, including the P1 and P2. Under normal physiological conditions, the waveform morphology reveals a pattern in which P1 > P2 > P3, which P1 represents the percussion wave related to the arterial blood pression maximum (Fig. 3-B), P2 represents the repercussion wave (tidal wave) that reflects cerebral compliance and was calculated as the middle point between the P1 maximum and the dicrotic notch (Fig. 3-B), and P3 that represents the closure of the cardiac aortic valve^18^. Changes in the waveform morphology may reveal pathological changes in the brain compliance, in which P2 > P1 represents reduced brain compliance^19–21^. Then, the P2/P1 ratio variable was also calculated (Fig. 3-C), considering the average of each individual over time.

### Statistical analysis

The sample size was based on a previous study using the same monitoring method proposed in the current study^17^ with a mean difference in the P2/P1 ratio of 0.30 (SD: 0.45) between groups which resulted in an effect size of 0.66. The power and the alpha level were set at 80% and 5% respectively resulting in a minimum of 29 participants in each group.

Independent samples t-test was used to compare age and body mass index between groups. The Analysis of Covariance was applied to compare the dependent variable P2/P1 ratio between groups by using the body mass index as a covariable. The normal distribution was confirmed by Shapiro–Wilk test. A Multiple Linear Regression was used to investigate the relationship between the P2/P1 ratio (dependent variable) and the following independent variables: body mass index, groups (with and without migraine), central sensitization, pain catastrophizing and depression, respectively in a hierarchical model, which the first block had the body mass index to control a covariable effect. Pearson and Spearman’s correlation coefficient were used to investigate the correlation between P2/P1 ratio and migraine frequency and migraine onset, pain intensity during attacks and pain intensity at day of examination, disability and allodynia in migraine group. The correlation was interpreted as low rs < 0.40), moderate (rs ≥ 0.40 to rs < 0.70), and high (rs ≥ 0.70). A significance level of 0.05 was established, and all analyses were performed using the SPSS software (version 17.0; SPSS Inc., Chicago, IL).

### Ethics approval and consent to participate

The Ethics Committee of the Clinics Hospital of Ribeirão Preto Medical School, University of São Paulo approved this study (protocol number: 1278/2019). All participants signed an informed consent form prior to data collection.

### Consent for publication

Informed consent to publish has been obtained to publish the information/image in an online open-access publication.

## Results

Women in both groups had on average 32 years old, once the control group was age-matched (Table 1). Women were evaluated on average of 10.93 days after the beginning of the menstrual cycle in the migraine group and 10.47 days in the control group, and two women in each group self-reported being in the perimenstrual phase. Women with migraine had a headache frequency of 12.3 (7.1) days per month and showed little (3.45%), mild (31.00%) and severe disability (65.52%) (Table 1). Regarding the type of medication, 51.7% of women in migraine group self-reported the use of analgesics, 41.3% of antidepressants, 24.1% of anti-inflammatory, 20.6% of antipsychotics, 6% of anxiolytics and 20.6% of antiepileptic drugs.

**Table 1:** Descriptive data which values are presented as mean (SD) and frequency (percentage).

	Migraine group (n = 29)	Control group (n = 29)
Age (years)	32.41 (11.16)	32.10 (9.00)
BMI (kg/m^2^)	24.74 (3.75)	22.78 (2.81)*
P2/P1 ratio	0.99 (0.24)	0.93 (0.18)
Pain catastrophizing (PCS)	23.71 (11.25)	11.80 (12.26)
Central sensitization (CSI-BR)	40.93 (15.71)	20.39 (8.18)
Depression (BDI)	14.67 (10.16)	4.62 (5.60)
Migraine frequency (monthly)	12.63 (7.45)	N/A
Episodic migraine, n (%)	18 (62.1%)	N/A
Chronic migraine, n (%)	11 (37.9%)	N/A
Migraine onset (years)	9.77 (6.90)	N/A
Pain intensity (NRS: 0 to 10)	7.79 (1.62)	N/A
Pain intensity at day	2.63 (2.56)	N/A
Presence of Aura, n (%)	13 (44.2%)	N/A
Disability (MIDAS)	43.50 (31.92)	N/A
Allodynia (ASC-12)	5.92 (2.83)	N/A

BMI, body mass index; NRS, numeric rating scale; MIDAS, Migraine Disability Assessment, ASC-12, 12-item allodynia symptom checklist; BDI, Beck Depression Inventory.

*p < 0.05 for independent samples t-test.

All descriptive data are presented at Table 1. The migraine group had higher body mass index (mean difference: 1.95, IC95%: 0.21 to 3.70, *p* = 0.03, t = 2.24). The Analysis of Covariance revealed no difference (F (1) = 0.568, *p* = 0.352) in the P2/P1 ratio comparison between the migraine (mean: 0.99, SD: 0.24) and control (mean: 0.93, SD: 0.18) groups (mean difference: 0.044, IC95%: -0.07 to 0.16) adjusted by the covariable body mass index (F (1,1) = 0.881, *p* = 0.352).

The Multiple Linear Regression analysis showed non-statistically significant model [F (5,44) = 1.104; *p* = 0.372; R^2^ = 0.11)] between the P2/P1 ratio and the presence of migraine, central sensitization, pain catastrophizing and depression, also corrected by the covariable body mass index.

We found no correlation between P2/P1 ratio and clinical data measured in migraine group: migraine frequency (r = -0.33, IC95% -0.55 to -0.07 , *p* = 0.08), migraine onset (r = 0.04, IC95% -0.26 to 0.30, *p* = 0.85), pain intensity (r = 0.12, IC95% -0.15 to 0.37 , *p* = 0.54), pain intensity at day (r = -0.03, IC95% -0.29 to 0.23, *p* = 0.86), disability (r = -0.17, IC95% -0.41 to 0.10, *p* = 0.39) and allodynia (r = 0.24, IC95% -0.02 to 0.47, *p* = 0.22).

## Discussion

This cross-sectional study found no differences between women with and without migraine in the waveform morphology, P2/P1 ratio through the ICP-NI measurement. Moreover, there was no association between the P2/P1 ratio and body mass index, central sensitization, pain catastrophizing and depression, and also no correlation with clinical variables such as migraine frequency, migraine onset, pain intensity and severity of allodynia.

In the context of existing knowledge, our results develop previous research findings that have investigated the role of ICP in patients with migraine migraine^5–7^ by using an innovative and non-invasive method of intra cranial pressure measurement^12,13^. Differently from our findings, abnormal waveforms have been suggested in migraine and chronic tension-type headache patients by an invasive and continuous monitoring, even in patients with normal opening pressure (< 200 mmH20), mainly in chronic migraine patients with bilateral transverse sinus stenosis diagnosed on cerebral magnetic resonance venography^5,6^. Chronic migraine patients have a high prevalence of transverse sinus stenosis, a marker of idiopathic intracranial hypertension^5^. Although idiopathic intracranial hypertension without papilledema associated with sinus stenosis is also prevalent in asymptomatic individuals, in migraineurs this may be related to migraine progression and chronification, possibly due to sensitization of trigeminovascular system pathways by the sinus congestion^5,7^.

Recent neuroimaging studies have suggested structural and functional brain alterations such as grey and white matter volume differences and metabolic changes in migraine patients^29^. Despite the recent advances, the authors highlight the relevance of exploring new biomarkers in order to better understand the migraine pathophysiology and possibly develop new treatment strategies^30^. Methods of non-invasive measurements of the intra cranial pressure, as the proposed in our study, support and increase the feasibility to better understand the central alterations of migraine patients. Our study reveals that patients with an average frequency of 12.6 attacks per month may not present changes in the intra cranial pressure. However, these findings expand the perspective of future studies considering different subtypes of migraine, which could assist the diagnosis and treatment choices in the future.

ICP waveform is determined by a complex interaction among the arterial input, intracranial tissues and the venous outflow^31^. Abnormalities in the ICP waveforms, configuration and amplitude, as an increased P2/P1 ratio, are evident in severe traumatic brain injuries^19^, due to changes in intracranial compliance and cerebral blood flow autoregulation. Recent evidences showed abnormalities in the ICP waveforms in different conditions by using the same non-invasive method as proposed in our study, through assessment of the volume changes in the skull, measured by an electric sensor in millivolts^32–35^. Alteration in the P2/P1 ratio were observed in hydrocephalus children^32^, in renal disease patients before and after dialysis^34^ and in case studies of children with untreatable headaches and secondary obstructive hydrocephalus^33^ and idiopathic intracranial hypertension^35^. On the other hand, no waveform abnormalities were seen in migraineurs in the current study, which represents a less severe disease but still involving a complex neurovascular dysfunction.

We also did not find correlation between the P2/P1 ratio and clinical variables, except by a weak positive correlation between increased P2/P1 ratio and higher pain catastrophizing considering women from both groups. Otherwise, previous finds suggest that fibromyalgia patients experienced symptoms relief as reduced headache and back pain, improved mood, concentration, quality of sleep and bowel and bladder function, up to 8 weeks after lumbar puncture^4^ even considering that about 50% of sample had opening pressure classified as normal (< 200 mmH2O). In this context, the cutoff point of 200 to 250 mmH2O to define the diagnosis of idiopathic intracranial hypertension has been discussed^3^, since also some pain conditions of unknown origin could be presented with an incomplete form or a variation of idiopathic intracranial hypertension^3^. Furthermore, high concentrations of anti-inflammatory cytokines as IL-1 receptor antagonist (IL-1ra) and proinflammatory cytokines as monocyte chemoattractant protein-1 (MCP-1)^36^ and interleukin-8^37^ have been found in the cerebrospinal fluid of fibromyalgia^37^ and migraine patients^36^ when compared to asymptomatic patients, although the role of inflammation in migraine is still uncertain^38,39^.

The present study has some weakness, such as the absence of an invasive method of monitoring the pressure in real values to further investigate between-groups differences considering the waveform morphology. On the other hand, the proposed method in our study has been previously validated^14,16^ in patients with traumatic and non-traumatic acute brain injury under invasive ICP monitoring. There was strong correlation for P2/P1 ratio between invasive and non-invasive monitoring and a cut-off 1.2 (P2/P1 ratio) predictive of intracranial hypertension (AUROC 0.9 for ICP > 20 mmHg)^14^. Our study did not include a cerebral magnetic resonance venography to investigate possible alterations such as transverse sinus stenosis to directly compare with previous literature^5,6^. Also, although we tried to control the menstrual cycle period for data collection, there were two women in each group in the perimenstrual period once the control group was age-matched. Furthermore, we did not control the migraine status (ictal, preictal, ictal) in the assessment day and some patients reported mild headache during the measurements. However, since migraine is a cyclic disease, patients might present physiological alterations even in the interictal phase^40^. Due the exploratory essence of our study – using a pioneer method to assess the intracranial pressure in migraineurs – we recommend that future studies consider not just the migraine phase, but also the migraine subdiagnosis in the evaluation. On the other hand, although our study has limitations, our findings may contribute to the literature on the hypotheses of changes in pressure of cerebrospinal and intracranial pressures in migraine patients by showing the applicability of a new method to extract parameters from ICF waveform. Accordingly, although the gold standard method is the intraventricular catheter system considering accuracy and reliability, other noninvasive ICP monitoring methods including brain imaging methods (magnetic resonance imaging, computed tomography), indirect ICP estimation (optic nerve sheath ultrasound, fundoscopy, etc.), cerebral blood flow evaluation (transcranial Doppler), and also the proposed in our study, represents an easy-to-measure and risk-free procedure^41^. Advances in new non-invasive methods and evidences of its application in different populations can benefit the applicability in further scientific studies and also in clinical practice of this methods as an alternative to invasive methods.

## Conclusion

Migraine patients did not present alterations in the waveform morphology through noninvasive intracranial pressure compared to women without headache. Also, there were no association between the P2/P1 ratio from this noninvasive measurement and clinical variables.

## Abbreviations

ICP-NI: Noninvasive intracranial pressure
CSF: Cerebrospinal fluid
ICP: Intracranial pressure
MIDAS: Migraine Disability Assessment
IL-1ra: IL-1 receptor antagonist
MCP-1: Monocyte chemoattractant protein-1
